# Ectopic Cushing’s syndrome associated with a pheochromocytoma in a dog: a case report

**DOI:** 10.1186/s12917-020-2244-7

**Published:** 2020-02-03

**Authors:** Sungin Lee, Aeri Lee, Suh-Hyun Chai, Seulji Lee, Oh-kyeong Kweon, Wan Hee Kim

**Affiliations:** Department of Veterinary Clinical Sciences, College of Veterinary Medicine and Research Institute for Veterinary Science, Seoul National University, 1 Gwanak-ro, Gwanak-gu, Seoul, 151-742 Republic of Korea

**Keywords:** Adrenal tumor, Dog, Ectopic Cushing’s syndrome, Hyperadrenocorticism, Pheochromocytoma

## Abstract

**Background:**

Ectopic Cushing’s syndrome (ECS) associated with malignant tumors, such as small cell lung carcinoma, bronchial carcinoids, and pheochromocytoma, has been reported in human medicine. However, ECS related to pheochromocytoma has not been reported in dogs.

**Case presentation:**

An 11-year-old castrated, male Scottish terrier was diagnosed with a left adrenal mass. Cushing’s syndrome was suspected based on clinical signs, including pot belly, polyuria, polydipsia, bilateral alopecia, recurrent pyoderma, and calcinosis cutis. Cushing’s syndrome was diagnosed on the basis of consistent clinical signs and repeated adrenocorticotropic hormone (ACTH) stimulation tests. In addition, tests for fractionated plasma metanephrine/normetanephrine suggested a pheochromocytoma. Unilateral adrenalectomy was performed after medical management with trilostane and phenoxybenzamine. Histopathology confirmed a diagnosis of pheochromocytoma without cortical lesions. After surgery, fractionated metanephrine/normetanephrine and the findings of low-dose dexamethasone suppression and ACTH stimulation tests were within the normal ranges without any medication. There were no clinical signs or evidence of recurrence and metastasis on thoracic and abdominal X-rays and ultrasonography up to 8 months after surgery.

**Conclusions:**

Pheochromocytoma should be considered a differential diagnosis for dogs with Cushing’s syndrome with an adrenal tumor. A good prognosis can be expected with prompt diagnosis and surgical intervention.

## Background

Ectopic Cushing’s syndrome (ECS) occurs when malignant tumors, including small cell lung carcinoma, bronchial carcinoids, pancreatic carcinoids, thymic carcinoids, and pheochromocytoma, produce adrenocorticotropic hormone (ACTH), ACTH precursors, or corticotropin releasing hormone (CRH) outside the pituitary glands [1–3]. Ectopic Cushing’s syndrome accounts for approximately 5% of all human cases of Cushing’s syndrome [3]. Primary hepatic carcinoid and neuroendocrine tumors are reportedly related to ECS; however, to the authors’ knowledge, canine ECS related to pheochromocytoma has not been previously reported [4–6]. In the present case study, we describe ECS caused by pheochromocytoma in a dog.

## Case presentation

An 11-year-old male, castrated Scottish terrier weighing 15.6 kg was examined at the Veterinary Medical Teaching Hospital of Seoul National University for a left adrenal mass and suspected Cushing’s syndrome. The owner reported a 3-month history of polyuria, polydipsia, abdominal distention, and recurrent pyoderma. Physical examination revealed a pot belly, bilateral alopecia, and pyoderma. In addition, calcinosis cutis was diagnosed on the basis of clinical assessments of the skin lesions as well as calcification observed in abdominal radiography. The dog displayed intermittent excitement and panting, with normal heart rate, respiratory rate, rectal temperature, and indirect systolic blood pressure. A complete blood count and serum biochemistry showed normal values for all parameters except elevations of alanine aminotransferase (451 U/*l*; reference range, 5.8–83.3 U/*l*), alkaline phosphatase (534 U/*l*; reference range, 0–97.9 U/*l*), and glucose (129 mg/d*l*; reference range, 74.5–120 U/*l*).

**Fig. 1:**
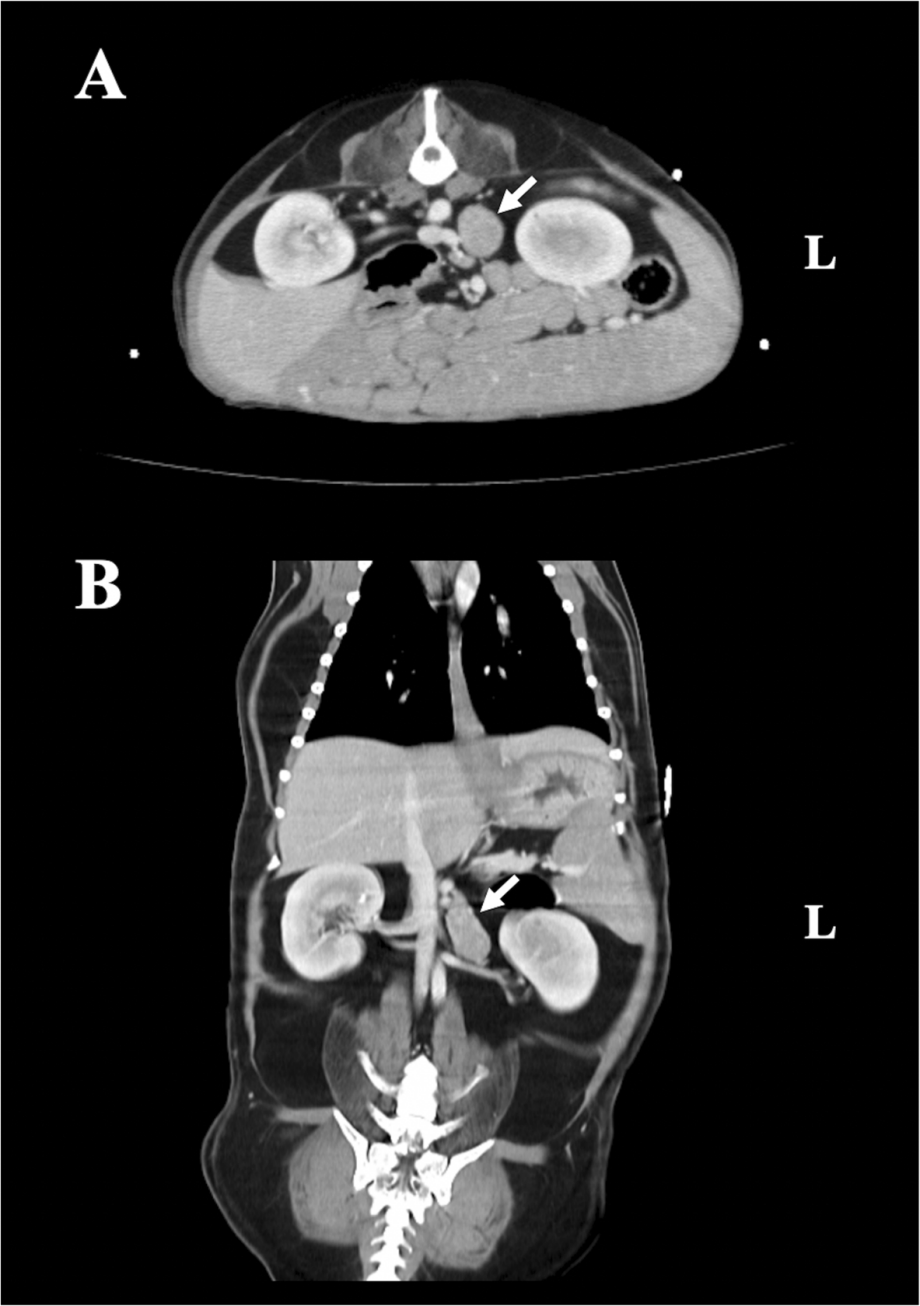
Abdominal computed tomography findings for a dog with ectopic Cushing’s syndrome associated with a pheochromocytoma. Transverse (**a**) and coronal (**b**) views show a left adrenal mass measuring 3.9 × 2.0 × 2.1 cm

Abdominal ultrasonography revealed asymmetrical enlargement of the left adrenal gland and a normal size and shape of the right adrenal gland (cranial pole, 0.56 cm; caudal pole, 0.50 cm). Abdominal computed tomography identified a left adrenal mass measuring 3.9 × 2.0 × 2.1 cm, with slightly heterogeneous enhancement and no invasion of adjacent vessels such as the caudal vena cava, aorta, and phrenicoabdominal vein (Fig. 1). The abovementioned imaging modalities showed no evidence of metastasis to the thoracic and abdominal organs.

The urinary corticoid:creatinine ratio, a screening parameter to rule out Cushing’s syndrome, was high (93.8; reference range, < 34). The ACTH stimulation test was done to confirm the diagnosis of Cushing’s syndrome. The basal cortisol level was 1.78 (reference range, 1–6 μg/d*l*) and post-ACTH cortisol level was 43.0 (reference range, 5.5–18 μg/d*l*) ug/d*l*. Cushing’s syndrome was diagnosed on the basis of consistent clinical signs and repeated hormonal blood analyses. Examinations for differentiating pituitary-dependent hyperadrenocorticism (PDH) from functional adrenocortical tumor (FAT) were performed. The serum endogenous ACTH level was 114.4 pg/m*l* (reference range, 10.0–100.0 pg/m*l*), while the high-dose dexamethasone suppression test (HDDST) revealed a basal serum cortisol level of 1.4 μg/d*l*, which was suppressed to < 0.5 μg/d*l* after 4 and 8 h. Fractionated free metanephrine (1.75 nmol/*l*; pheochromocytoma reference range, 1.0–102.0 nmol/*l*) and normetanephrine (7.34 nmol/*l*; pheochromocytoma reference range, 3.3–211.0 nmol/*l*) levels in the plasma suggested the possibility of a pheochromocytoma [7]. Because the results of ultrasonography and HDDST were not consistent (HDDST results suggested PDH; unilateral enlargement of the adrenal gland suggested FAT), PDH and FAT could not be clearly distinguished. After consulting with the owner, surgery was performed to remove the suspected pheochromocytoma involving the left adrenal gland.

Before surgery, a urine dip stick test indicated proteinuria (2+) with a protein:creatinine ratio of 3.10. This was managed by oral telmisartan (1 mg/kg) once daily and oral enalapril (0.5 mg/kg) twice daily. In addition, twice-daily oral trilostane (1 mg/kg) and phenoxybenzamine (0.5 mg/kg) were prescribed. Preoperative trilostane treatment to reduce the risks of anesthesia relieved the clinical signs of Cushing’s syndrome and resulted in normal ACTH levels. Left adrenalectomy was performed under dexamethasone treatment to decrease the possibility of hypoadrenocorticism after removal of the adrenal mass. Postoperative recovery was uneventful, however, hypocortisolism was observed in an ACTH stimulation test performed immediately after surgery. Prednisolone was administered for 4 weeks, following which the medications were withheld for 24 h before repetition of the ACTH stimulation test; the values were within the reference range. The clinical signs of Cushing’s syndrome gradually decreased after surgery. A low-dose dexamethasone suppression test performed at 4 months showed normal values (0 h, 1.7 μg/d*l*; 4 h, < 0.5 μg/d*l*; 8 h, < 0.5 μg/d*l*) without any medication. Plasma fractionated metanephrine (0.49 nmol/*l*; normal reference range, 0.3–1.2 nmol/*l*) [7], normetanephrine (1.90 nmol/*l*; normal reference range, 0.9–2.1 nmol/*l*) [8], and serum endogenous ACTH (11.2 pg/m*l*; reference range, 10.0–100.0 pg/m*l*) levels normalized after surgery. There were no clinical signs or evidence of recurrence and metastasis on thoracic and abdominal X-rays and ultrasonography up to 8 months after surgery.

**Fig. 2:**
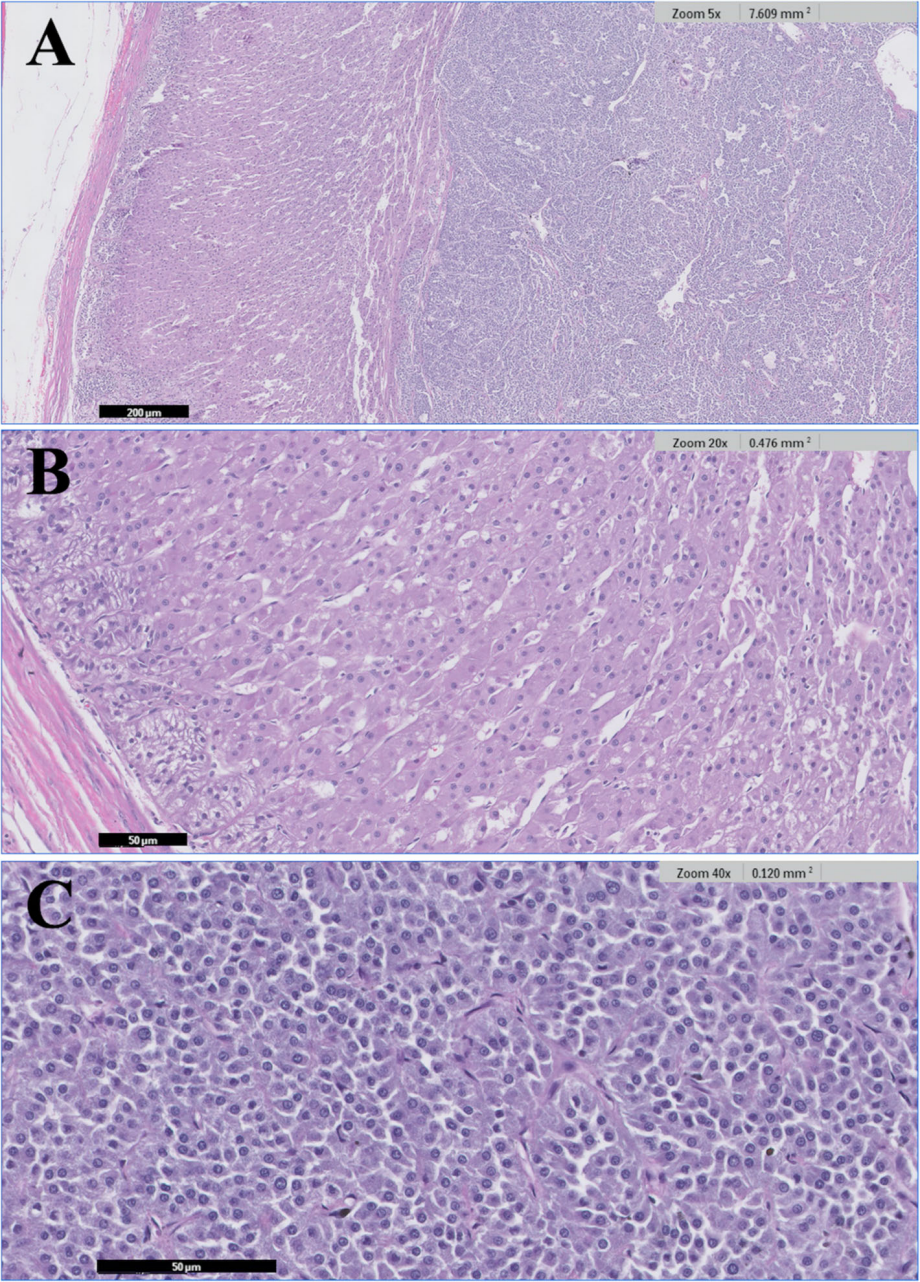
Histopathology of a left adrenal mass without adrenal cortical lesions in a dog with Cushing’s syndrome. The mass was diagnosed as pheochromocytoma. (**a**) Image showing both the cortex and the tumor. (**b**) Magnified image of the cortex. (**c**) Magnified image of the tumor

**Fig. 3:**
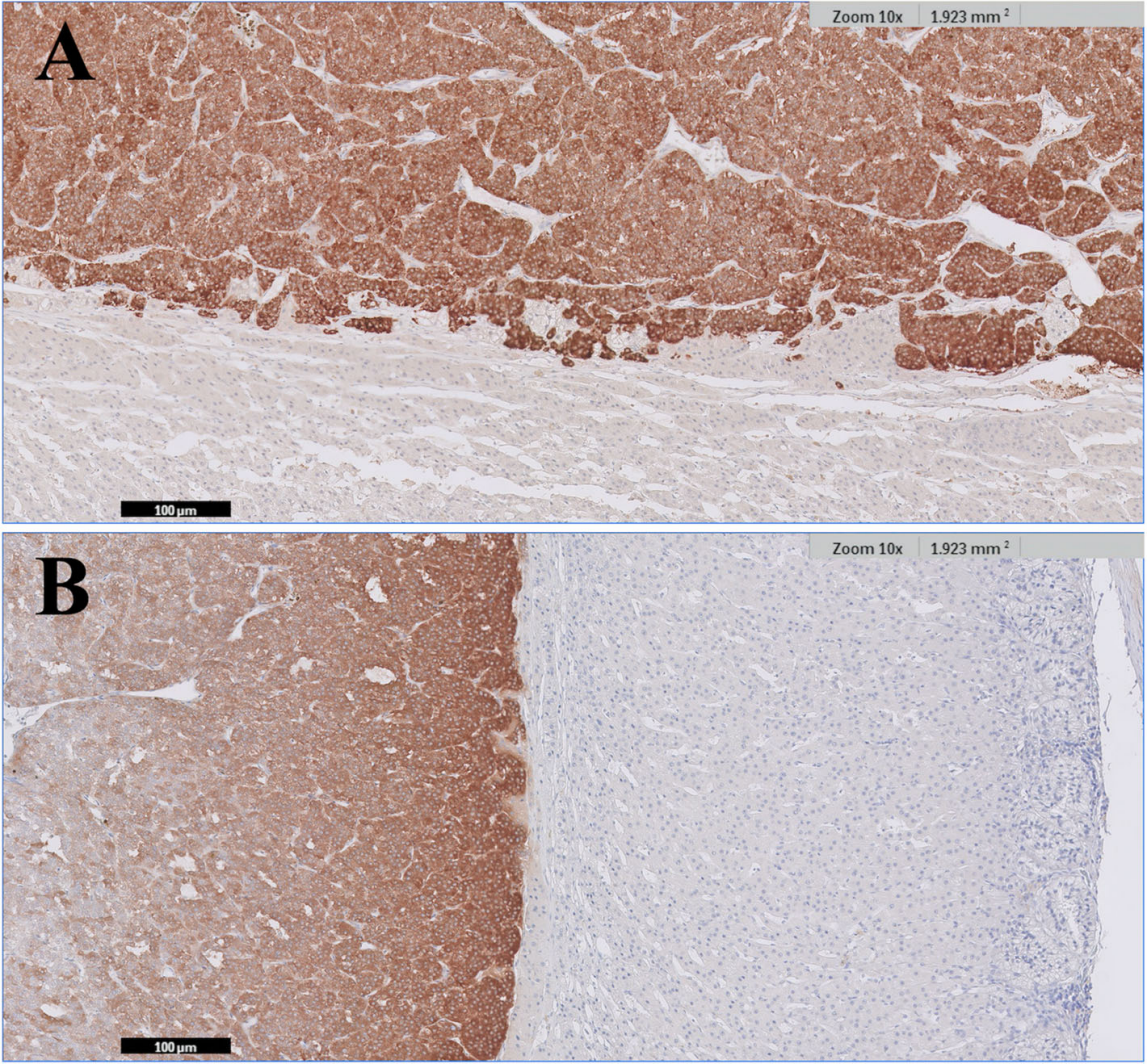
Immunohistochemical examinations for chromogranin A and synaptophysin in the pheochromocytoma and adrenal cortex. The neoplastic cells show strong and diffuse positive staining. The adrenal cortex shows negative staining. (**a**) Chromogranin A. (**b**) Synaptophysin

**Fig. 4:**
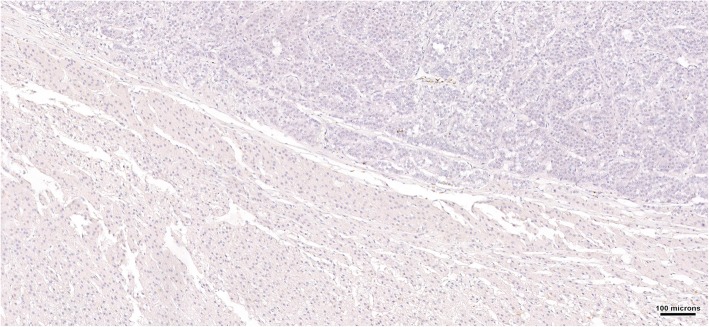
Immunohistochemistry for adrenocorticotropic hormone (ACTH) in the pheochromocytoma and adrenal cortex. The findings are negative; the neoplastic cells show no evidence of positive staining

The resected adrenal mass was processed with 10% formaldehyde immediately after surgery and referred for histopathological analysis the following day. Histopathological analysis of the adrenal mass confirmed a diagnosis of pheochromocytoma without any adrenal cortical lesions, including hyperplasia (IDEXX Laboratories, Inc., USA) (Fig. 2). Immunohistochemical examinations for chromogranin A and synaptophysin showed positivity in the neoplastic mass (IDEXX Laboratories, Inc., USA) (Fig. 3). Immunohistochemistry for ACTH revealed negative findings (IDEXX Laboratories, Inc., USA) (Fig. 4).

## Discussion and conclusions

Cushing’s syndrome, one of the most commonly diagnosed endocrine disorders in dogs, is characterized by hypercortisolism. Common causes include PDH, characterized by the excessive pituitary production of ACTH, and FAT, characterized by autonomous cortisol secretion from the adrenocortical tumor [9]. Ectopic production of CRH and ACTH has been reported infrequently in humans and rarely in dogs. To the authors’ knowledge, ECS caused by pheochromocytoma in dogs has not been previously reported, although previous case reports involving canines with mesenteric neuroendocrine tumors and a primary hepatic carcinoid suggested that the tumors were related to ECS [4–6]. A case involving a 5-year-old male Dachshund with Cushing’s syndrome was reportedly due to ectopic secretion of ACTH by an unidentified microadenoma. The authors did not find evidence of an ACTH-producing lesion in any gland, including the pituitary gland, on imaging and postmortem examinations [10].

Pheochromocytoma, a catecholamine-producing tumor involving the chromaffin cells in the adrenal medulla, is uncommon in dogs and rare in cats [8, 9, 11]. The expression of clinical manifestations, including panting, tachypnea, weakness, collapse, lethargy, inappetence, and hypertension, is dependent on the secretion of catecholamines such as epinephrine and norepinephrine by the tumor. However, because catecholamine release by the tumor is typically episodic, the clinical signs may be intermittent, thus complicating its diagnosis [9, 12, 13]. Canine pheochromocytoma is generally considered malignant. In the literature related to veterinary medicine, there are no reports of chemotherapy and radiation for canine pheochromocytoma; therefore, surgical removal is considered the most appropriate definitive treatment [9]. In dogs with pheochromocytoma, the administration of alpha-adrenoreceptor antagonists such as phenoxybenzamine before adrenalectomy helps in preventing mortality during anesthesia and surgery. A previous retrospective study showed that the mortality rate for dogs that received phenoxybenzamine before surgery was 3.7-fold lower than that for dogs that did not receive the treatment [14]. In the present case, a pheochromocytoma was suspected and phenoxybenzamine was administered before surgery, which was uneventfully completed.

Although there are no case reports of ECS caused by a pheochromocytoma in dogs, human cases have been documented, related to ectopic secretion of ACTH, CRH, or ACTH precursors [15–17]. A previous mini-review/case study showed that the clinical signs of Cushing’s syndrome and elevated catecholamine and ACTH levels normalized after removal of the affected adrenal gland. The resected adrenal mass was diagnosed as a pheochromocytoma, with positive immunohistochemical staining for ACTH confirmed in approximately 40% neoplastic cells [18]. In addition, the patient fulfilled the diagnostic criteria for ACTH-secreting pheochromocytoma (modified by Chen et al. in 1995), including: (1) clinical and laboratory evidence of hypercortisolism, (2) elevated plasma ACTH levels, (3) biochemical or imaging evidence of a pheochromocytoma, (4) resolution of symptoms and signs of adrenocorticoid and catecholamine excess after unilateral adrenalectomy, and (5) rapid normalization of plasma ACTH levels after adrenalectomy. The patient was definitively diagnosed with an ACTH-secreting pheochromocytoma [18, 19]. An ectopic CRH-secreting pheochromocytoma was identified by immunohistochemistry for CRH, and an ectopic ACTH precursor-secreting pheochromocytoma was diagnosed by measuring ACTH precursors and observation of secretion of ACTH precursors in cultured tumor cells, which decreased after the in vitro application of dexamethasone [16, 17].

In the present case, preoperative trilostane treatment reduced the risks of anesthesia, decreased the clinical signs of Cushing’s syndrome, and normalized the ACTH stimulation test results. Before surgery, Cushing’s syndrome was confirmed on the basis of clinical signs, biochemical analysis, and responses on trilostane therapy; left unilateral adrenalectomy resulted in normalization of clinical signs and biochemical tests, without medications. The resected adrenal mass was confirmed by histopathology to be a pheochromocytoma without any cortical lesions, including hyperplasia. In general, cases of ECS do not exhibit suppression in HDDST because the adrenal axis is already inhibited. In the present case, however, suppression was observed in HDDST. In previous studies, approximately 30% patients with ECS showed serum cortisol suppression in HDDST [3, 20, 21]. Moreover, because 20–33% cases of ECS were reportedly misdiagnosed because of suppressed cortisol levels, the use of other modalities such as the urinary cortisol:cortisone ratio, pituitary magnetic resonance imaging (MRI), and Gallium-68-somatostatin receptor positron emission tomography/computed tomography (PET/CT) was suggested to improve the diagnostic accuracy [22–24]. After comprehensive analysis, the possibility of PDH was ruled out, and the final diagnosis was ECS associated with a pheochromocytoma.

To further investigate the association of the pheochromocytoma with hypercortisolism, we performed immunohistochemistry for ACTH, which showed negative findings for the neoplastic cells. In previous reports of a pituitary ganglioma and mesenteric neuroendocrine tumor, the presence of ectopic ACTH syndrome was confirmed by positive immunohistochemistry for adrenocorticotropin in the surgically resected tumors [6, 25]. In contrast, some studies have reported cases of ectopic ACTH syndrome, although they did not show positive immunostaining for ACTH in the neoplastic tissue [4, 5, 26, 27]. Cases involving a 69-year-old woman and 43-year-old man with an ectopic ACTH-secreting pheochromocytoma also showed negative immunostaining for ACTH. The authors suggested that negative staining was probably due to a CRH-secreting tumor, small ACTH-derived peptides, or ACTH precursors with a high molecular weight that could not be detected by the antibodies used for immunohistochemistry [28, 29]. Therefore, the possibility of ectopic ACTH syndrome or ECS associated with the secretion of CRH or ACTH precursors cannot be ruled out in the present case.

The present case study has some limitations. Although the association between Cushing’s syndrome and pheochromocytoma has been shown, the underlying mechanism was not established. Several diagnostic modalities, including perioperative ACTH precursors and CRH, urinary cortisol:cortisone ratio, pituitary MRI, and Gallium-68-somatostatin receptor PET/CT to evaluate the locations and the release of ACTH or CRH from the adrenal mass, could be useful to investigate the relationship between ECS and pheochromocytoma.

To the author’s knowledge, this is the first case report of ECS associated with pheochromocytoma in dogs. Pheochromocytoma should be considered a differential diagnosis in dogs with Cushing’s syndrome and adrenal tumors. A good prognosis can be achieved with prompt and appropriate diagnosis, preoperative management, and surgical intervention.

## Data Availability

All relevant data are within this paper. The datasets generated during the current case study are available from the corresponding author on reasonable request.

## Abbreviations

ACTH: Adrenocorticotropic hormone
CRH: Corticotropin releasing hormone
ECS: Ectopic Cushing’s syndrome
FAT: Functional adrenocortical tumor
HDDSL: High-dose dexamethasone suppression test
PDH: Pituitary-dependent hyperadrenocorticism
